# Creatine Transporter Defect Diagnosed by Proton NMR Spectroscopy in Males With Intellectual Disability

**DOI:** 10.1002/ajmg.a.34208

**Published:** 2011-09-09

**Authors:** Maria Antonietta Mencarelli, Maria Tassini, Marzia Pollazzon, Antonio Vivi, Marco Calderisi, Michele Falco, Marco Fichera, Lucia Monti, Sabrina Buoni, Francesca Mari, Udo Engelke, Ron A Wevers, Joussef Hayek, Alessandra Renieri

**Affiliations:** 1 Medical Genetics, Department of Biotechnology, University of SienaSiena, Italy; 2 Medical Genetics, Azienda Ospedaliera Universitaria SeneseSiena, Italy; 3 The NMR Center University of SienaSiena, Italy; 4 Laboratory of Genetic Diagnosis, IRCCS Associazione Oasi Maria SantissimaTroina, Italy; 5 Unit of Diagnostic and Therapeutic Neuroradiology and Inter Departmental Center of Nuclear Magnetic Resonance, Azienda Ospedaliera SeneseSiena, Italy; 6 Child Neuropsychiatry, Azienda Ospedaliera SeneseSiena, Italy; 7 Laboratory of Genetic Endocrine and Metabolic Diseases, Department of Laboratory Medicine, Radboud University Nijmegen Medical CentreNijmegen, The Netherlands

**Keywords:** creatine deficiency syndrome, *SLC6A8*, magnetic resonance spectroscopy, NMR urine

## Abstract

Creatine deficiency syndrome due to mutations in X-linked *SLC6A8* gene results in nonspecific intellectual disability (ID). Diagnosis cannot be established on clinical grounds and is often based on the assessment of brain creatine levels by magnetic resonance spectroscopy (MRS). Considering high costs of MRS and necessity of sedation, this technique cannot be used as a first level-screening test. Likewise, gene test analysis is time consuming and not easily accessible to all laboratories. In this article feasibility of urine analysis (creatine/creatinine (Cr/Crn) ratio) performed by nuclear magnetic resonance (NMR) as a first level-screening test is explored. Before running a systematic selection of cases a preliminary study for further molecular analysis is shown. NMR urine spectra (n = 1,347) of male patients with an ID without a clinically recognizable syndrome were measured. On the basis of abnormal Cr/Crn ratio, three patients with the highest values were selected for molecular analysis. A confirmatory second urine test was positive in two patients and diagnosis was further confirmed by a decreased brain creatine level and by *SLC6A8* gene analysis. A de novo mutation was identified in one. Another patient inherited a novel mutation from the mother who also has a mild ID. A repeat urine test was negative in the third patient and accordingly creatine level in the brain and *SLC6A8* gene analysis both gave a normal result. We conclude that Cr/Crn ratio measured by NMR for male patients represents a rapid and useful first level screening test preceding molecular analysis. © 2011 Wiley-Liss, Inc.

## INTRODUCTION

Creatine deficiency syndrome caused by *SLC6A8* gene mutations was first described in 2001 by Salomons et al. [2001]. These authors characterized the presence of a mutation in the X-linked *SLC6A8* gene in one patient with intellectual disability (ID) with absence of creatine in brain MRS and increased creatine in urine and plasma. In 2004, the same group assessed the frequency of this condition in a group of 288 males with an X-linked pedigree with at least two affected males [Rosenberg et al., 2004]. By gene sequence analysis, they found that *SLC6A8* mutations account for 2% of X-linked ID males (6/288). In 2006, the same group investigated the frequency of this condition among ID sporadic cases [Clark et al., 2006]. In a total of 478 ID males by gene sequence analysis they found 4 pathogenic mutations concluding that the condition may have at least a frequency of 0.8% among ID males. Afterwards, a lower prevalence (0.3%) was observed in a cohort of 1,600 patients with ID of unknown origin, previously selected by HPLC–MS/MS [Arias et al., 2007]. In 2009 an Estonian group explored the frequency of the condition in 49 familial ID case with a compatible X-linked transmission by measuring the creatine/creatinine (Cr/Crn) ratio in urine [Puusepp et al., 2009]. The authors found eight false positive families and one *SLC6A8* mutated family confirming the frequency of 2% among X-linked ID families. Mercimeck-Mahmutoglu et al. [2009] developed a stable isotope dilution tandem mass-spectrometry method to determine the Cr/Crn ratio in 975 individuals. They confirmed a *SLC6A8* mutation in 2 males among 87 with one or more symptoms at risk for *SLC6A8* defects, giving a frequency of *SLC6A8* deficiency of 2.3% in the cohort at risk.

Since the first description, more than 60 index cases affected by this disorder and a total of 150 patients have been ascertained [Stöckler-Ipsiroglu and Salomons, 2006]. The clinical phenotype ranges from mild ID and speech delay to severe ID, seizures, and behavioral disorders. The diagnosis cannot be established on clinical grounds only as the clinical signs and symptoms are rather aspecific and the suspicion of the disease is based on the assessment of brain creatine level by MRS or by measurement of the Cr/Crn ratio in urine [Almeida et al., 2004]. We have explored the feasibility of NMR spectroscopy of the urine to this end and included 258 male urine samples in our study.

In this study 258 urine NMR spectra of male children were studied. We selected three male patients on the basis of their Cr/Crn ratio and found that two have a pathogenic molecular defect in creatine transporter gene *SLC6A8*.

## MATERIALS AND METHODS

### Patients

A cohort of 258 males with ID was included in the medical study. Among these, 231 were sporadic cases, while 27 had at least one affected brother. All patients were evaluated by a clinical geneticist (AR, MAM, or MP) and clinically recognizable syndromes were excluded. In all cases molecular analysis of *FMR1* gene resulted normal.

### Urine NMR Analysis

Urine ^1^H-NMR measurements were carried out on a 600 MHz Bruker DRX Avance Spectrometer with a Selective Inverse Probe (SEI) equipped with Z gradient coil. Spectra were acquired at the constant temperature of 298.0 ± 0.1 K using a 90° pulse. A 10 sec delay was included in the pulse sequence to allow T_1_ relaxation as previously described [Engelke et al., 2009]. Suppression of the water signal was achieved by applying a saturation pulse of 2 sec duration at the water resonance. 32K data points per scan were used and 128 transients were accumulated. The total experimental time for each spectrum was 27 min. Each urine sample was measured after centrifugation at 2,000 rpm for 5 min. Sample (550 µl) plus 50 µl of a TSP-d_4_ 20 mM solution, were transferred into a 0.5 mm (outer diameter) NMR tube at pH 2.50 ± 0.02. The chemical shift of ionizable substances is highly dependent on pH. At pH 2.50 all chemical shift values are reproducible within ±0.01 ppm [Lehnert and Hunkler, 1986]. Moreover, under these conditions, the methyl signals of creatine and creatinine are clearly separated (3.05 ppm for the methyl signal of creatine and 3.13 ppm for the methyl signal of creatinine). The pH adjustment to 2.50 was the only sample pretreatment. The pH was adjusted with a minimal volume of HCl, starting from a 3 M and ending with a 0.05 M solution (dilution effect less than 5%). The pH meter used was model 420A Orion connected with a MI-412 combination electrode from Microelectrodes, Inc., Bedford, New Hampshire. The Cr/Crn ratio could be measured directly from the areas of the signals without any additional quantification. Statistical calculations using a bootstrap resampling procedure have been carried out using “R”, a free software environment for statistical computing and graphics (R Development Core Team, 2010).

### Molecular Analysis of the *SLC6A8* Gene

The 13 exons and the consensus splice sites of the *SLC6A8* gene were analyzed. A total of eight PCR reactions were performed: exons 5-6-7, 8-9-10, and 11-12, were sufficiently close to be amplified as three fragments. Primers and PCR conditions are available upon request. PCR products were purified with ExoSAP-IT (USB Corporation, Cleveland, OH) and bidirectionally sequenced on an ABI3130 Genetic Analyser (Applied Biosystems, Foster City, CA) using the same PCR primers.

### Brain MRI and ^1^H MRS

Patients underwent 1.5 T magnetic resonance imaging (MRI) and magnetic resonance spectroscopy (^1^H MRS) examinations. A multivoxel technique with SE sequences at TE 30 msec and TE 135 msec was used. The multivoxels ^1^H MRS 2D technique was acquired on the centrum ovale. All voxels examined in the patients had normal appearing brain tissue on conventional MRI examination. The local magnetic field homogeneity is automatically optimized with an auto-shim procedure and adjusted for chemical-shift-water suppression. ^1^H-MRS raw data were transferred to a Leonardo Workstation, reconstructed and processed by Siemens software (Erlangen, Deutschland). Peak areas of choline (Cho), creatine (Cr), and *N*-acetyl aspartate (NAA) were estimated by numeric integration. Metabolite signals were measured from single spectra. White matter intravoxel (Cr/Cho, Cho/Cr, and NAA/Cr) ratios were calculated from the peak areas.

## RESULTS

### Urine Proton NMR Spectroscopy

**FIG. 1:**
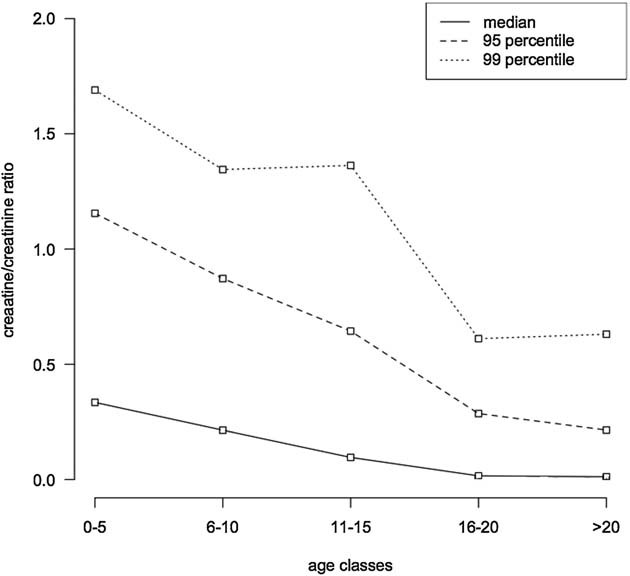
Median, 9th and 99th centile for each age classes as a result of the above-described bootstrap technique starting from the Cr/Crn ratios of 1,347 urine samples.

**FIG. 2:**
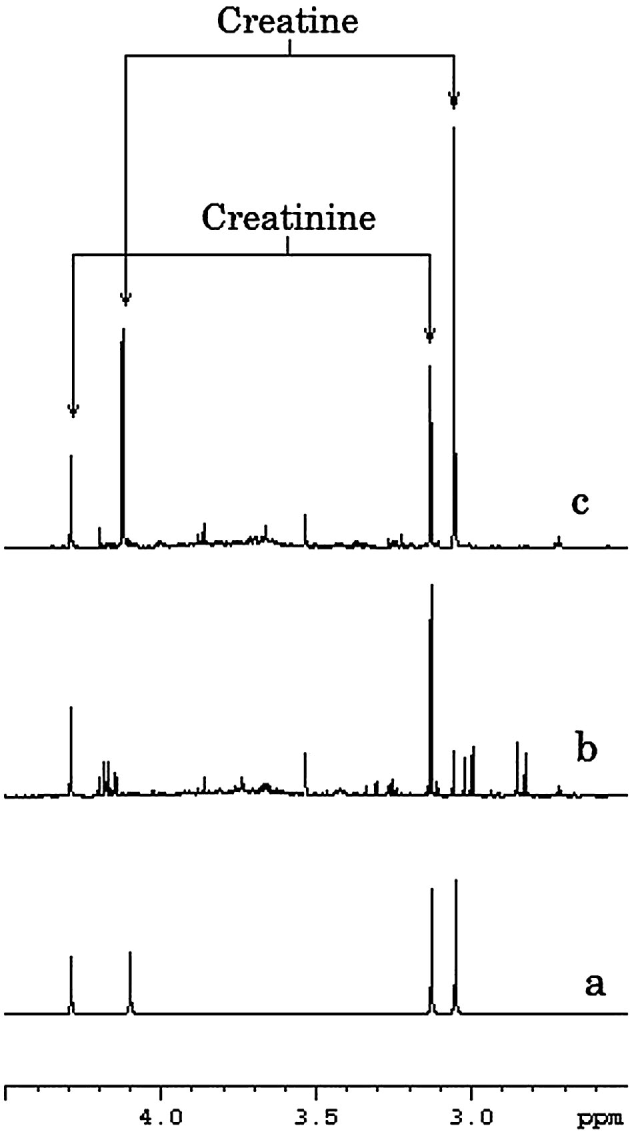
Proton NMR spectra measured at pH 2.50. **a**: Standard 0.1 M solution of pure substances creatine (3.05 and 4.11 ppm) and creatinine (3.13 and 4.29 ppm). We can clearly see the separation between the signals: a singlet at 3.05 ppm from the methyl of creatine and a singlet at 3.13 ppm from the methyl of creatinine. The two other singlets come from the methylene of creatine (at 4.11 ppm) and creatinine (at 4.29 ppm). **b**: Urine spectrum of a healthy subject. **c**: Urine spectrum of a patient affected by creatine transporter defect. The arrows indicate the creatine and creatinine signals.

As no age- and sex-related reference values are available for the Cr/Crn ratio in urine we derived reference intervals (p5, 50, and 95) from our total patient group. The data did not show a Gaussian distribution and this was also not the case after logarithmic transformation. To obtain the reference intervals shown in Table I a bootstrap resampling procedure was used for data of which the distribution is not known. The dataset was split in age group classes (0–5, 6–10, 11–15, 16–20, and >20 years) (Fig. 1). Within each class we performed bootstrap resampling (n = 1,000). Bootstrap statistical techniques are computer intensive methods falling in the resampling techniques used as an alternative based parametric statistic (*p* < 0,05). Adèr et al. [2008] recommended the use of bootstrapping procedures when the theoretical distribution of a statistic is complicated or unknown. As the bootstrapping procedure is distribution-independent, it provides an indirect method to assess the properties of the distribution underlying the sample and the parameters of interest. There was an obvious age and sex relation of the data. Subsequently the three male patients with the highest Cr/Crn ratio were selected. The ratio was consistently abnormal in two patients but two repeat samples in patient three showed normal results (Fig. 2; Table I).

**TABLE I:** Creatine/Creatinine Ratios Measured by NMR Urine Spectroscopy in Male Patients, Related *SLC6A8* Gene Mutations and Age Classes With the Related 5, 50, 95 Percentiles as Calculated After Bootstrap Resampling

Patient	NMR Cr/Crn urine ratio	Age at the time of sample collection (years)	Age classes (years)	p5	p50	p95	*SLC6A8* mutation
1	2.080	14.0	11–15	0.008	0.096	0.648	c.C993G p.N331K
	3.350	15.5	11–15	0.008	0.096	0.648	
	2.500	16.5	16–20	0.004	0.016	0.275	
	2.300	17.5	16–20	0.004	0.016	0.275	
2	2.090	7.0	6–10	0.008	0.214	0.870	c.C1171T p.R391W
	2.120	7.5	6–10	0.008	0.214	0.870	
	1.880	8.5	6–10	0.008	0.214	0.870	
	1.590	12.5	11–15	0.008	0.096	0.648	
3	3.360	5.0	0–5	0.010	0.333	1.155	No mutations
	0.640	10.0	6–10	0.008	0.214	0.870	
	0.120	10.0	6–10	0.008	0.214	0.870	

### Clinical Description of the Patients With the Highest Cr/Crn Ratio

**FIG. 3:**
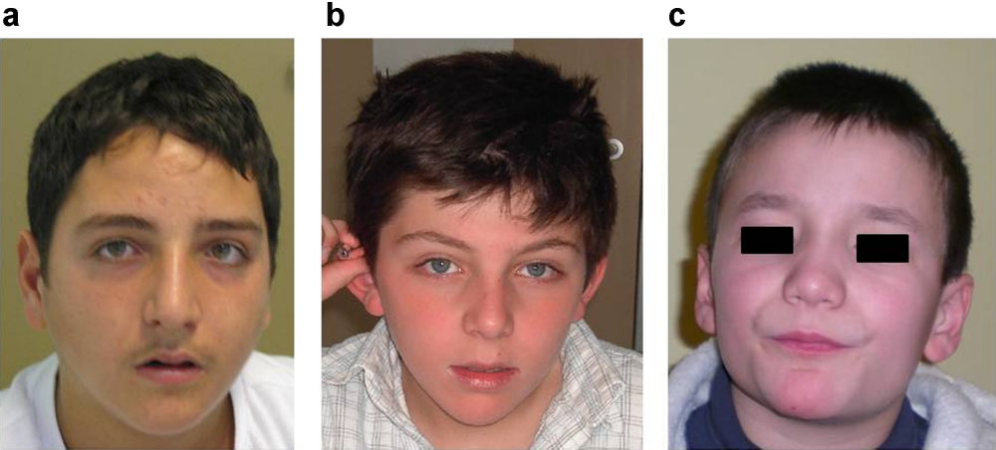
Photographs of Patient 1 (**a**), Patient 2 (**b**) and Patient 3 (**c**).

Patient 1 (#1115) (Fig. 3a) is a 18-year-old boy, the third child of nonconsanguineous and healthy parents. His mother showed a mild ID. He was born at term by caesarian after a pregnancy of normal course. At birth he showed a weight of 3,030 g (25–50th centile) and hypotonia. Psychomotor delay was noted since early infancy: crawling was acquired after 1 year and first single words after 1 year. He had two episodes of seizures, one of which in fever. Karyotype with resolution of 400 bands and molecular analysis of *FMR1*, *SCN1A*, and *GABRG2* genes were normal. Physical examination at 16 years and 8 months showed: height 174 cm (50–75th centile), weight 83 kg (90–97th centile), OFC 57 cm (90–97th centile), sparse eyebrows, long ears (7 cm, >+2DS), M-shaped upper lip, open mouth, normal hand- and foot length. He was not able to formulate sentences and showed hyperactivity, hyperphagia, and aggressiveness.

Patient 2 (#726) (Fig. 3b) is a 12-year-7 month-old boy, first child of nonconsanguineous and healthy parents. He was born at term by caesarian after a pregnancy of normal course. His birth weight was 3,500 g (50th centile). He showed speech delay and three episodes of myoclonic seizures. Psychometric evaluation at 11 years recorded an IQ of 50. Array-CGH analysis (105 K) was normal. Physical examination at 11 years and 6 months showed: height 138 cm (25th centile), weight 35 kg (50th centile), OFC 54 cm (75th centile), triangular face, and long ears. His language was simple and he showed hyperactivity and abnormal behavior.

### Brain MRS

**FIG. 4:**
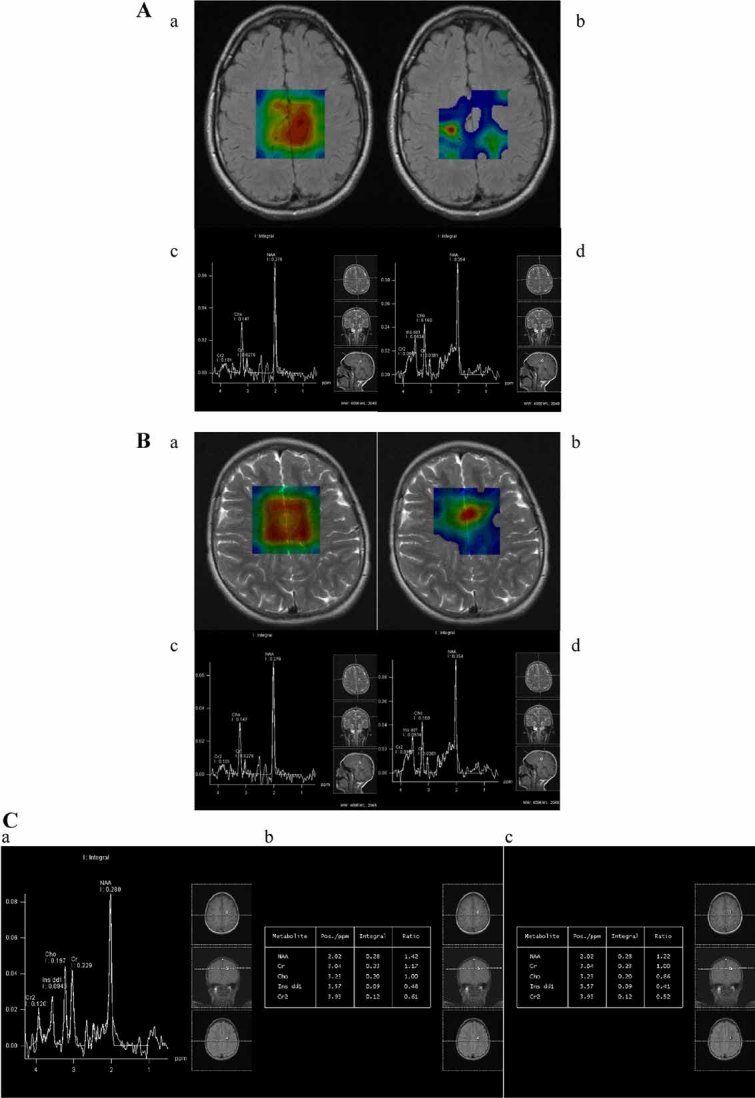
Panel (**A**) Patient 1's MRS spectra acquired with technique with SE sequences at TE 30 msec and TE 135 msec show severe reduction of the Cr peak. Red indicates a high concentration, while blue indicates a low concentration of metabolite. In (**a**) the concentration of NAA is reported. In (**b**) the Cr color map demonstrates only a small area with normal Creatine concentration. Tables of metabolite ratios (**c**,**d**). Panel (**B**) Axial FLAIR image of Patient 2 demonstrates normal appearing of white and gray matter in the centrum semiovale. An example of the two dimensional MRS sequence: data are presented as metabolic images of *N*-acetyl aspartate (NAA) (**a**) and creatine (Cr) (**b**). Red indicates a high concentration, while blue indicates a low concentration of metabolite. The Cr color map demonstrates a diffuse low concentration of creatine while the NAA color map shows a normal concentration of NAA. MRS technique with SE sequence at TE 30 msec demonstrates that the spectrum is severely abnormal with a Cr peak virtually absent. Tables of metabolite ratios (**c**,**d**). Panel (**C**) Normal MRS spectra of Patient 3 (**a**,**b**), comparable to a healthy 10 years old child (**c**).

In Patients 1 and 2 the results of Cho/Cr and NAA/Cr measurements in the white matter of centrum ovale demonstrated a significant abnormally high ratio compared to age matched healthy controls (Fig. 4). For Patient 1 the following ratios were found: Cho/Cr = 4.2, NAA/Cr = 12.8 at TE of 30 msec; Cho/Cr = 4.15 and NAA/Cr = 12.7 at TE of 135 msec. In Patient 2 these amounted to Cho/Cr = 4.7 and NAA/Cr = 9.3 at TE of 30 msec; Cho/Cr = 4.2 and NAA/Cr = 9.2 at TE of 135 msec. Patient 3 showed normal Cho/Cr and NAA/Cr ratios. In a control group (subjects, n = 10) at TE of 30 msec, the mean of Cho/Cr is 1.00 and the SD is 0.26; the mean of NAA/Cr is 2.21 and the SD is 0.35.

### Molecular Analysis of *SLC6A8* Gene

*SLC6A8* gene analysis revealed two missense mutations: in Patient 1 a novel mutation was identified (NM_005629.3:c.993C>G p.Asn331Lys) that was inherited from his mother who has a borderline IQ. Skewed X-inactivation was excluded. The mutation lies in a cytoplasmic domain and affects a residue which is highly conserved in different species (including *Mus musculus* BC141067, *Rattus norvegicus* NW_001084770, *Bos taurus* NM_174611, and *Oryctolagus cuniculus* NM_001082397). In Patient 2 a de novo mutation (NM_005629.3:c.1171C>T p.Arg391Trp, known mutation-PMID17465020: Rosenberg 2007) was identified. The mutation lies in a extracellular domain and affects a residue which is highly conserved in different species. Molecular analysis of *SLC6A8* gene showed no mutation in Patient 3 (Fig. 3c).

## DISCUSSION

Since its first description in 2001, approximately 150 patients have been reported in the literature with a diagnosis of X-linked creatine transporter deficiency. The low number of reported cases is definitely an underestimation of the frequency of this condition, since present data suggest that *SLC6A8* mutations will have a 3–5 times greater frequency than most other X-linked ID genes [Clark et al., 2006]. The frequency of *SLC6A8* mutations seems to be comparable to that of *FMR1* mutations and thus *SLC6A8* may be considered a major contributor to ID in the male population [Clark et al., 2006].

The few diagnosed patients reveal that this condition is not recognizable from a clinical point of view. Aspecific clinical phenotypes also occur in other X-linked ID-related diseases. However, creatine levels in biological fluids represent a strong diagnostic hint for this condition and this metabolite should be investigated in order to improve the diagnostic yield. Brain MRS, demonstrating creatine depletion, is the golden standard for the diagnosis. However, it is an expensive analysis not available in many hospitals and it is an invasive diagnostic tool due to the need of sedation. It thus cannot be considered a first level-screening test. Therefore, it would be important to use a rapid and less expensive test to select patients for further diagnostic analyses.

Urine screening has been employed in at least three studies using gas chromatography/mass spectrometry or LC/mass spectrometry. In 2007, Arias et al. [2007] determined Cr/Crn ratio in urine samples from 1,600 male patients with ID and/or autism and identified 4 true positive cases (0.3%) and 21 false positive cases (1.8%). More recently, in a study by Puusepp et al. [2009] 49 ID familial cases were preliminary selected for gene analysis by urine creatine assessment. In this study, the biochemical testing showed 18% false positives. In the study of Mercimeck-Mahmutoglu et al. [2009] the Cr/Crn ratio was determined by tandem mass-spectrometry leading to the identification of 2 true positive cases out of 29 individuals (6.9% false positives). In the present work we applied a biochemical screening test, by proton NMR spectroscopy measuring urine samples from 258 mostly sporadic ID males. We analyzed the Cr/Crn ratio followed by *SLC6A8* gene analysis in the three patients with the highest ratio. In two patients a pathogenic mutation was found confirming the suspected creatine transporter defect. The increased ratio in the third patient was most likely caused by dietary interference. This illustrates that a single urine sample is not enough to be sure of this diagnosis, so we recommend to attest an increased Cr/Crn ratio in at least two samples of a patient prior to more expensive and invasive testing. Genetic analysis is advised when the Cr/Crn ratio is consistently abnormal in almost two samples. It has the advantage to estimate the recurrence risk of the deficit for all family members. Our work demonstrates that urine NMR spectroscopy can be used to identify patients with a creatine transporter defect. It has the advantage that only little sample-pretreatment is needed (the pH adjustment). NMR spectroscopy will be also able to find cases with GAMT deficiency (guanidinoacetate methyltransferase) [Tassini et al., 2010]. The analysis can also be performed in frozen samples, which can be collected in peripheral hospitals and sent to referral hospitals later.

We have found a consistently abnormal Cr/Crn urine ratio in two patients and confirmed the decreased creatine concentration in brain of these patients. Results presented here and those previously published by Clarke et al. [2006] stress that a significant number of males with ID may have an *SLC6A8* mutation. Even though in our series of familial cases we did not identify any positive patient, present data suggest that the prevalence of *SLC6A8* mutations is even higher in familial cases reaching a 2% [Rosenberg et al., 2004; Puusepp et al., 2009].

An a posteriori re-evaluation of the clinical phenotype of positive patients confirmed that it is not specific. Both patients in whom we detect a *SLC6A8* mutation present moderate ID, mostly related to speech and language, hyperactivity, behavioral disturbances, and no dysmorphic features. Therefore, we suggest to perform urine NMR as a first level-screening test in males with ID regardless of the family history and to proceed to *SCL6A8* molecular analysis, if a high Cr/Crn ratio is consistent.
